# Author Correction: Embedded nano spin sensor for in situ probing of gas adsorption inside porous organic frameworks

**DOI:** 10.1038/s41467-023-41233-6

**Published:** 2023-09-04

**Authors:** Jie Zhang, Linshan Liu, Chaofeng Zheng, Wang Li, Chunru Wang, Taishan Wang

**Affiliations:** 1 Beijing National Laboratory for Molecular Sciences, Key Laboratory of Molecular Nanostructure and Nanotechnology, Institute of Chemistry, Chinese Academy of Sciences, Zhongguancun North First Street 2, Beijing, 100190 China; 2 University of Chinese Academy of Sciences, Beijing, 100049 China

**Keywords:** Chemistry, Carbon nanotubes and fullerenes

Correction to: *Nature Communications* 10.1038/s41467-023-40683-2, published online 15 August 2023

The original version of this article contained an error in Fig. 4a, in which label between state II and state III incorrectly read “Adsorb N_2_” rather than the correct “Adsorb C_3_H_6_”. This has been corrected in both the PDF and HTML versions of the article.

