# Supplementary material for: Embedded nano spin sensor for in situ probing of gas adsorption inside porous organic frameworks
Source: Nat Commun. 2023 Aug 15;14:4922. doi: 10.1038/s41467-023-40683-2 (PMC10427628; doi:10.1038/s41467-023-40683-2)
Supplement: Supplementary file 1 — Supplementary information [file 41467_2023_40683_MOESM1_ESM.pdf]

Supplementary information

Embedded nano spin sensor for in situ probing of gas adsorption inside porous organic frameworks

Jie Zhang et al.

Supplementary Note 1. Mass fraction of $Sc_3C_2@C_{80}$ in Py-COF

Toluene solution of $Sc_3C_2@C_{80}$ with a concentration of 4×10^{-5} mol·L^{-1} (2 ml) was prepared, and the mass of $Sc_3C_2@C_{80}$ was 8.952×10^{-2} mg. The solution was then analyzed by HPLC with a Buckyprep-M column to obtain a HPLC profile, and the sample was recycled. Then 2 mg of Py-COF with high crystallinity was immersed into the toluene solution of $Sc_3C_2@C_{80}$, and the $Sc_3C_2@C_{80}\subset$Py-COF complex was obtained by complete adsorption for one week. The remaining $Sc_3C_2@C_{80}$ solution was then analyzed by HPLC to obtain a profile, see Supplementary Fig. 2b. From the area difference before and after adsorption of $Sc_3C_2@C_{80}$, the content of $Sc_3C_2@C_{80}$ absorbed by Py-COF was calculated to be about 3×10^{-3} mg. Therefore, the mass fraction of $Sc_3C_2@C_{80}$ in Py-COF is 1.5‰.

Supplementary Note 2. EPR test of $Sc_3C_2@C_{80}\subset$Py-COF under different conditions

The sensing measurements were executed on an electronic paramagnetic resonance spectrometer. The vacuum treatment was performed for 30 min before each test. The gases were filled into the quartz tube and kept for 30 min.

Supplementary Note 3. EPR test of $Sc_3C_2@C_{80}$⊂Py-COF with the same adsorption amount

EPR tests were performed for the same adsorbed amounts of N_2, CO, CH_4, CO_2, C_3H_8, and C_3H_6 at different pressures of 1 bar, 0.79 bar, 0.45 bar, 0.12 bar, 0.03 bar, and 0.04 bar, respectively. Each condition was repeated three times to obtain the corresponding EPR spectra.

Supplementary Note 4. Mass fraction of $Sc_3C_2@C_{80}$ in MOF-177

Toluene solution of $Sc_3C_2@C_{80}$ with a concentration of 4×10^{-5} mol·L^{-1} (1 ml) was prepared, and the mass of $Sc_3C_2@C_{80}$ was 4.476×10^{-2} mg. The solution was then analyzed by HPLC with a Buckyprep-M column to obtain a HPLC profile, and the sample was recycled. Then 10 mg of MOF-177 with high crystallinity was immersed into the toluene solution of $Sc_3C_2@C_{80}$, and the $Sc_3C_2@C_{80}\subset$MOF-177 complex was obtained by complete adsorption for one week. The remaining $Sc_3C_2@C_{80}$ solution was then analyzed by HPLC to obtain a profile, see Supplementary Fig. 14b. From the area difference before and after adsorption of $Sc_3C_2@C_{80}$, the content of $Sc_3C_2@C_{80}$ absorbed by MOF-177 was calculated to be about 3.78×10^{-2} mg. Therefore, the mass fraction of $Sc_3C_2@C_{80}$ in MOF-177 is 3.78‰.

Supplementary Figures

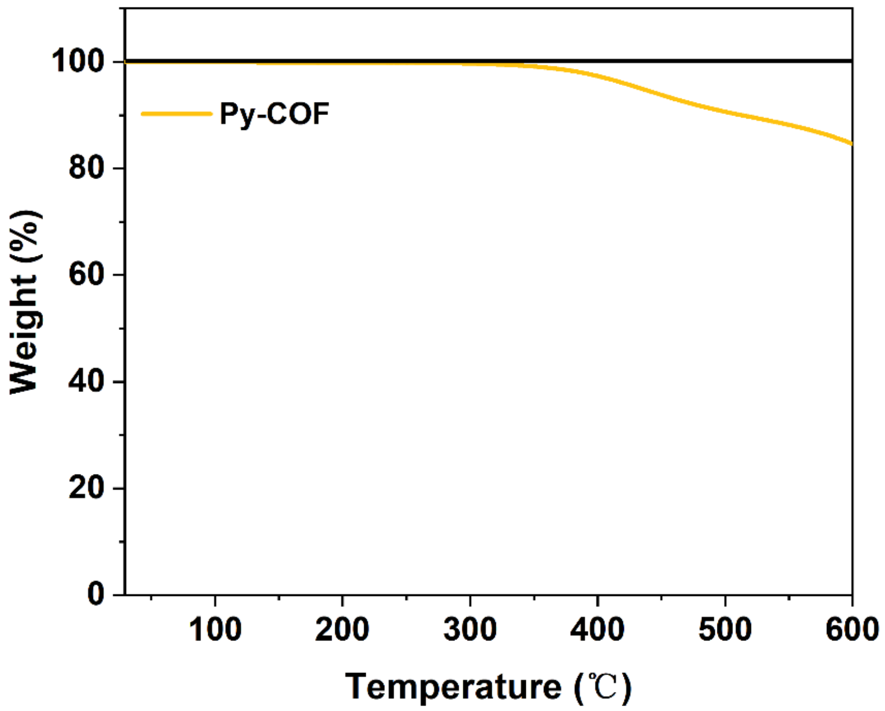

Supplementary Fig. 1 TGA curve for Py-COF powder.

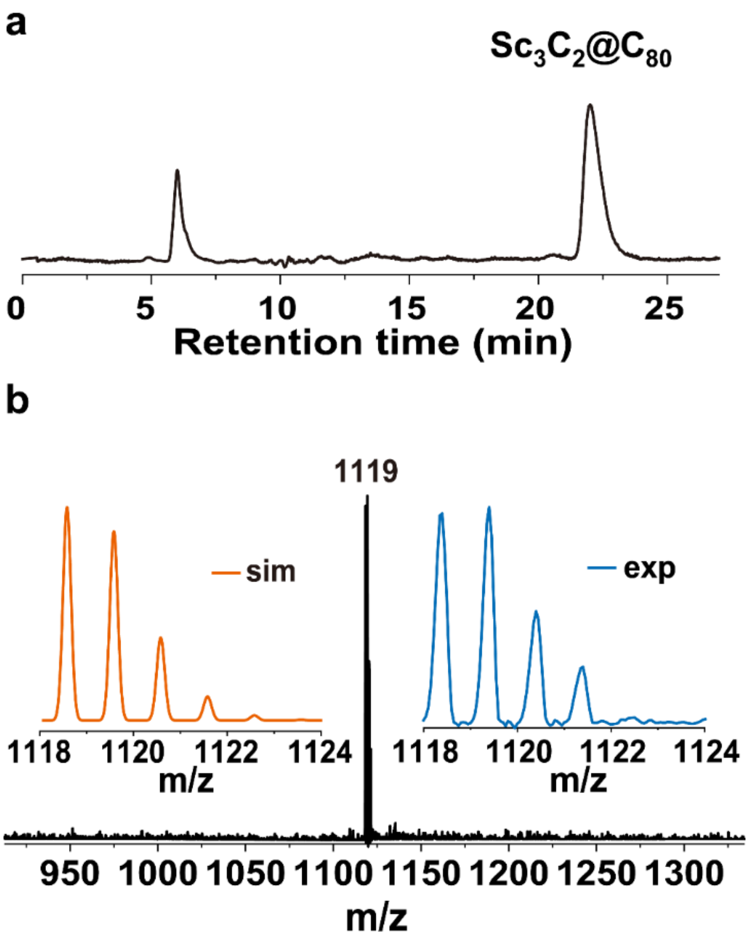

Supplementary Fig. 2 a Chromatogram of the isolated $Sc_3C_2@C_{80}$ (20×250 mm Buckyprep-M column; flow rate 12 mL/min; toluene as eluent). **b** MALDI-TOF MS profile of the isolated $Sc_3C_2@C_{80}$. The insets show the experimental and simulated isotope distribution patterns.

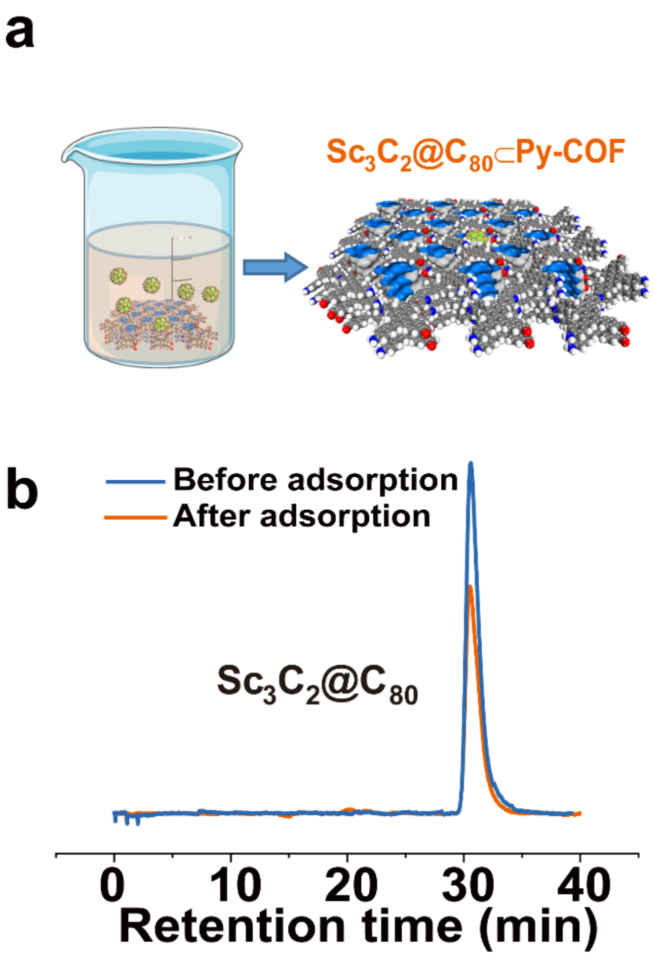

Supplementary Fig. 3 **a** The preparation process of $Sc_3C_2@C_{80}{\subset}$Py-COF. **b** HPLC chromatograms of the $Sc_3C_2@C_{80}$ solutions before and after adsorption by Py-COF. Figure created with BioRender.com.

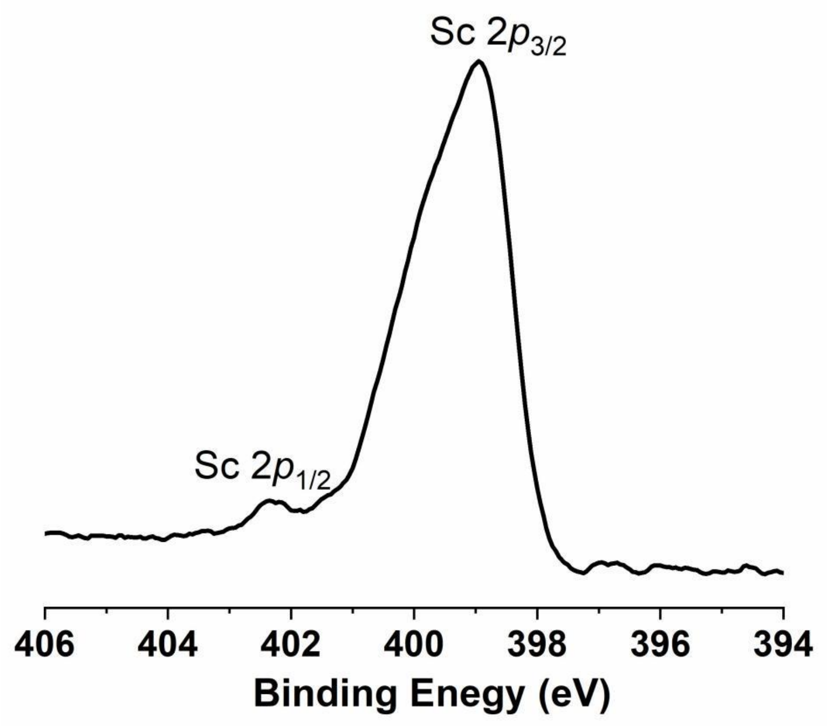

Supplementary Fig. 4 XPS spectrum of Sc 2*p* peak of $Sc_3C_2@C_{80}$⊂Py-COF. The peak positions of $2p_{3/2}$ and $2p_{1/2}$ are 398.95, 402.4 eV, respectively.

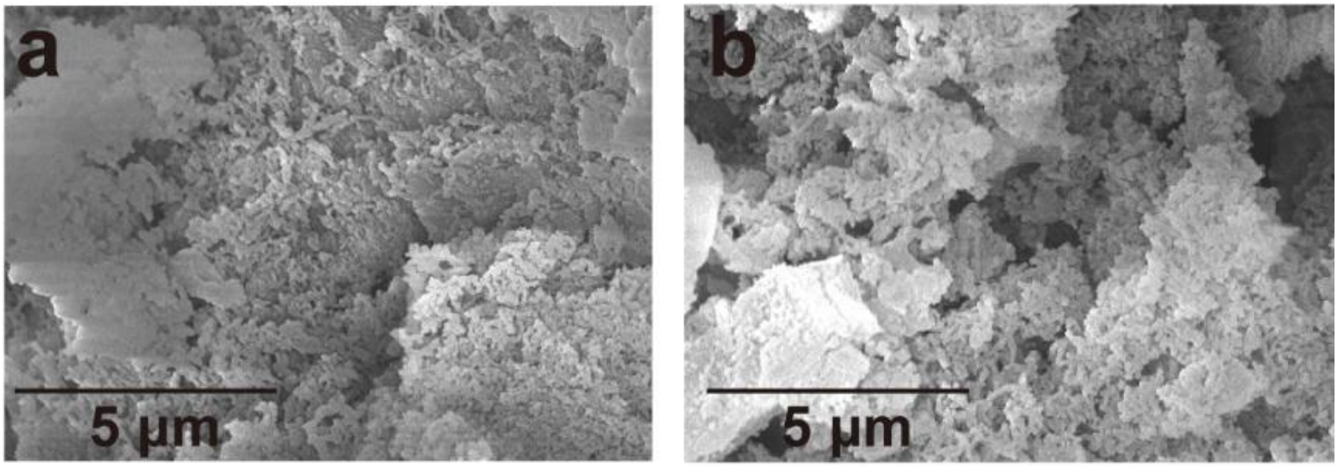

Supplementary Fig. 5 SEM image of **a** Py-COF and **b** $Sc_3C_2@C_{80}$⊂Py-COF complex.

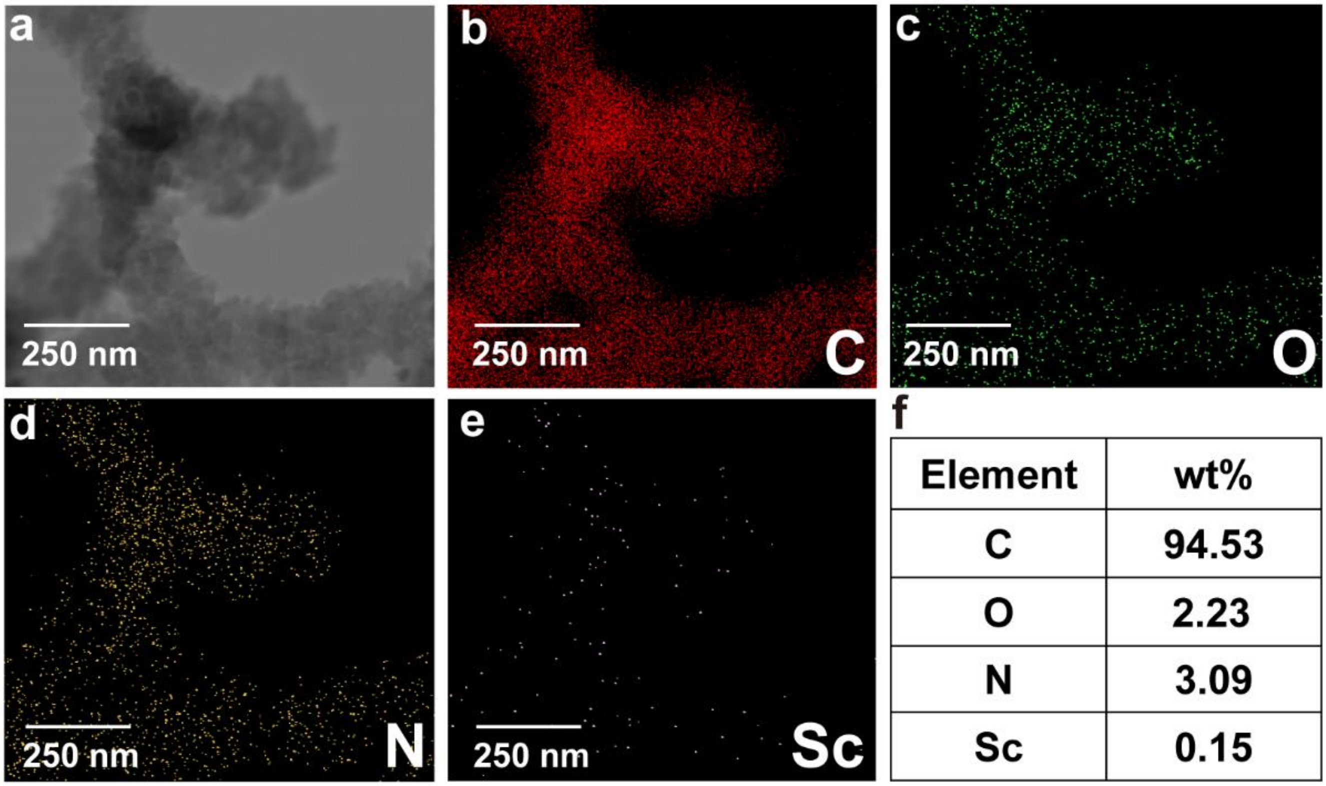

Element	wt%
C	94.53
O	2.23
N	3.09
Sc	0.15

Supplementary Fig. 6 TEM images of **a** $Sc_3C_2@C_{80}$⊂Py-COF complex. TEM element mapping images of **b** C, **c** O, **d** N and **e** Sc for $Sc_3C_2@C_{80}$⊂Py-COF complex. **f** Elemental fractions of $Sc_3C_2@C_{80}$⊂Py-COF from EDS spectrum.

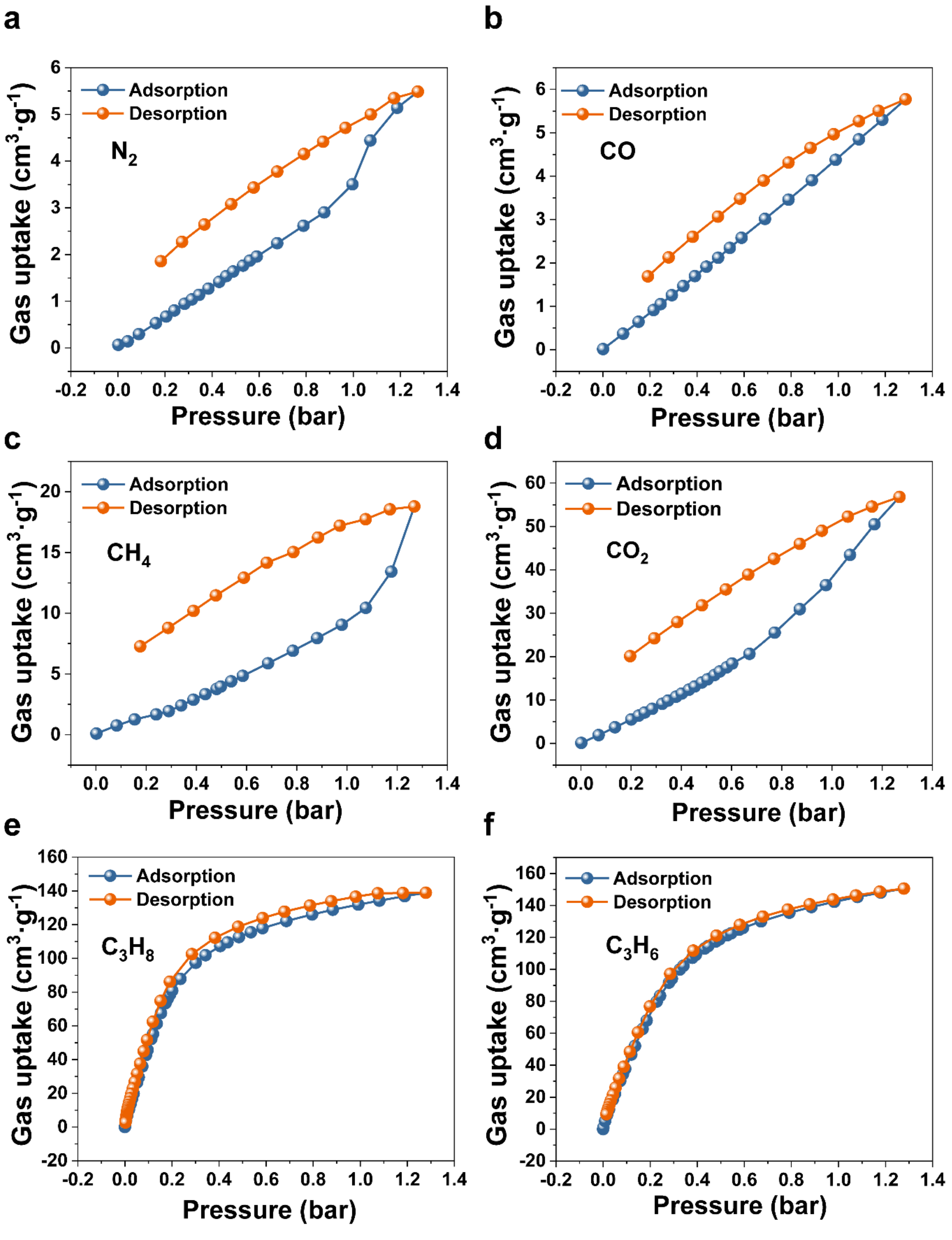

Supplementary Fig. 7 Adsorption and desorption isotherms of Py-COF for **a** N_2, **b** CO, **c** CH_4, **d** CO_2, **e** C_3H_8, and **f** C_3H_6.

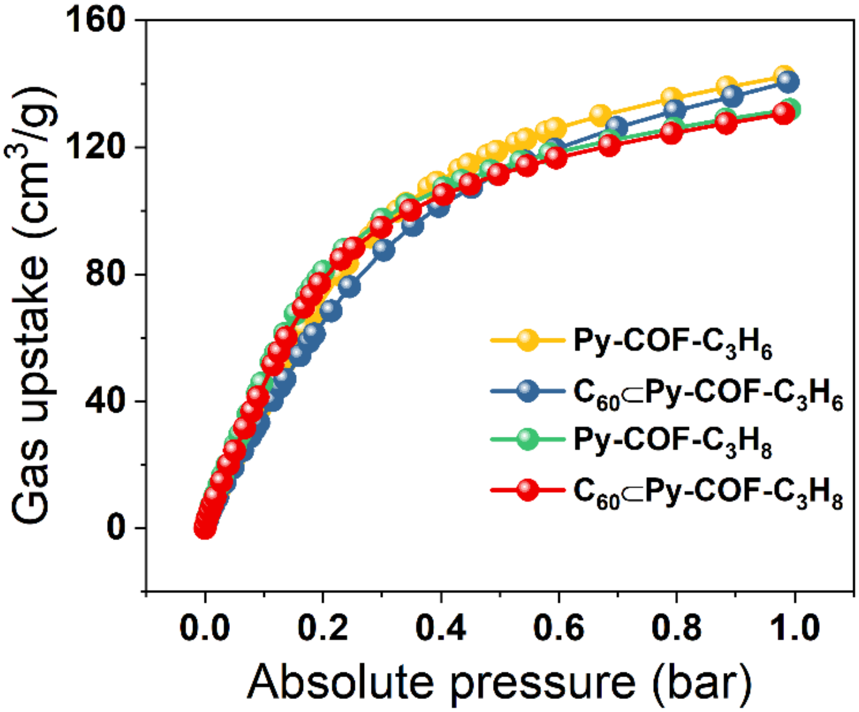

Supplementary Fig. 8 Measured adsorption isotherms of Py-COF and C_{60}⊂Py-COF (mass fraction: 9.8‰) for the C_3H_6 and C_3H_8 gases.

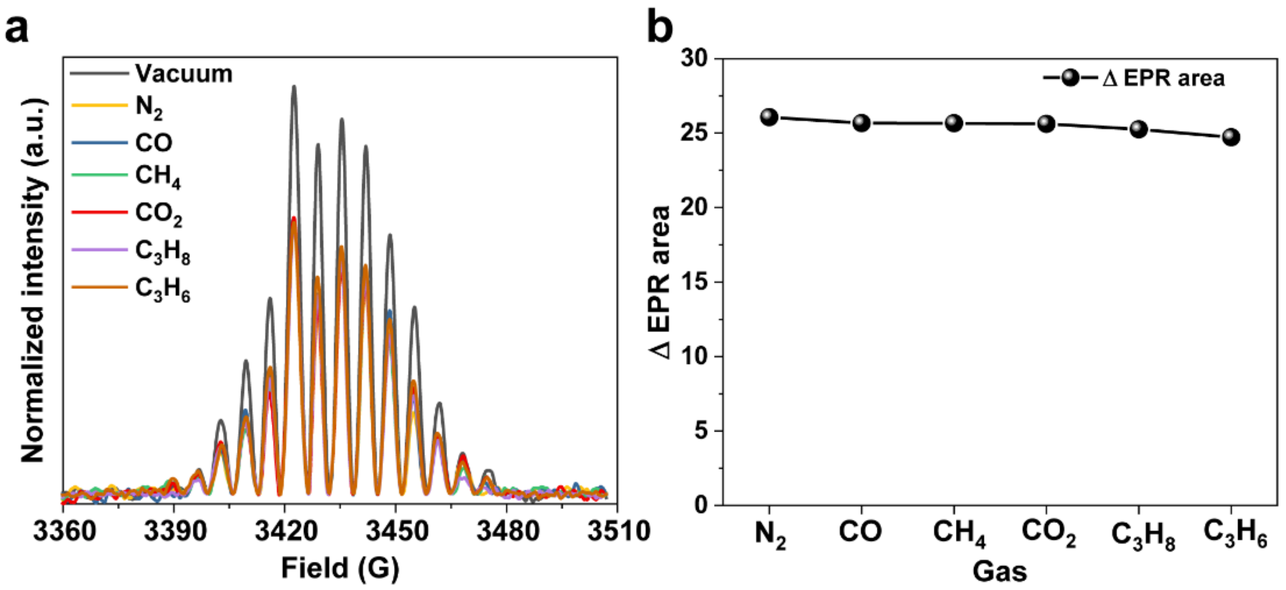

Supplementary Fig. 9 a Integrated EPR spectra of $Sc_3C_2@C_{80}$⊂Py-COF under different gas conditions with the same adsorption amount. **b** Integrated EPR signal areas for different gases at the same adsorption amount. EPR tests were performed for the same adsorbed amounts of N_2, CO, CH_4, CO_2, C_3H_8, and C_3H_6 at different pressures of 1 bar, 0.79 bar, 0.45 bar, 0.12 bar, 0.03 bar, and 0.04 bar, respectively.

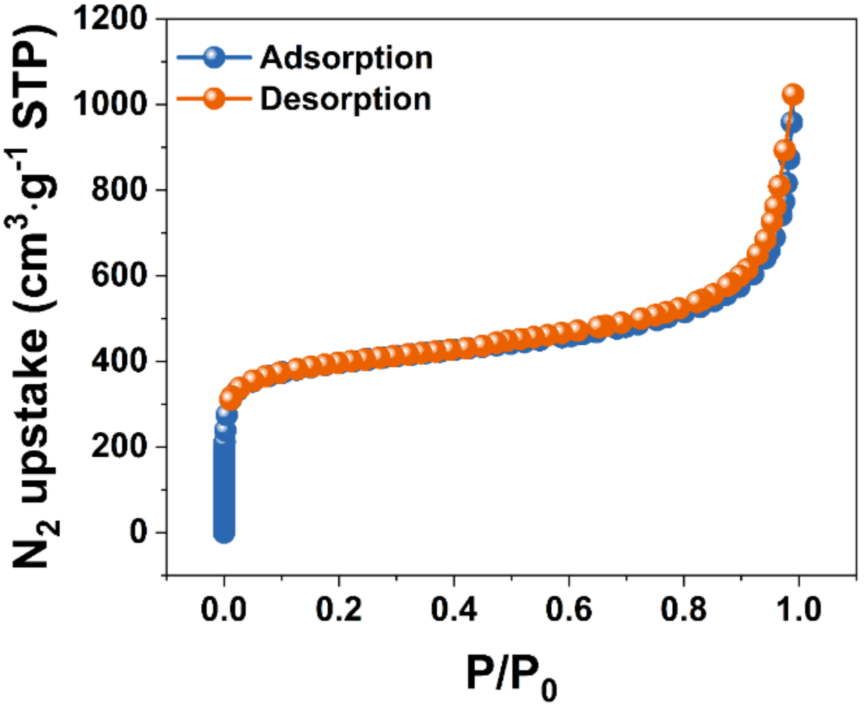

Supplementary Fig. 10 Adsorption and desorption isotherms of Py-COF for N_2 at 77 K.

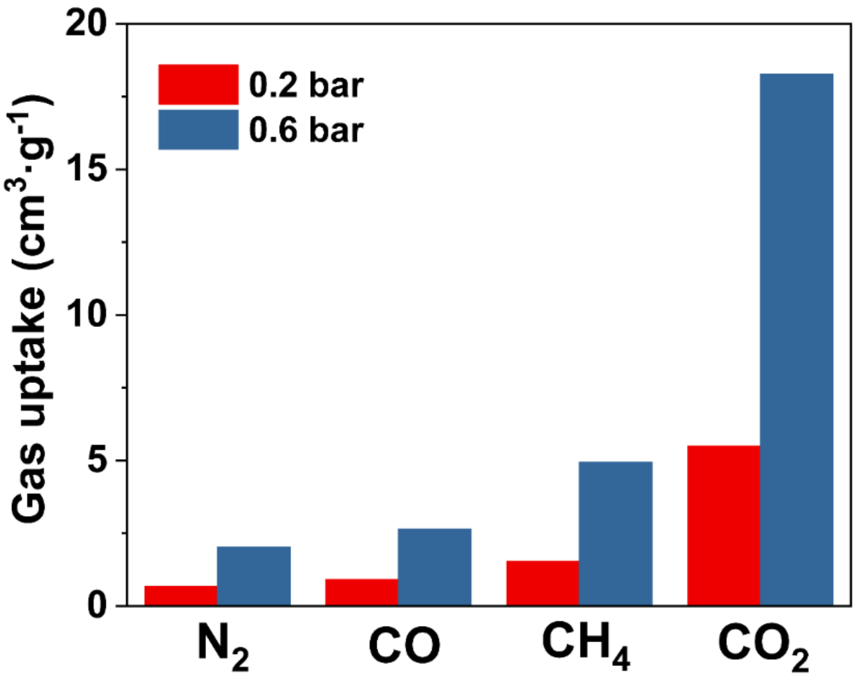

Supplementary Fig. 11 Measured adsorption amounts of different gases for Py-COF using adsorption isotherms at 0.2 bar and 0.6 bar.

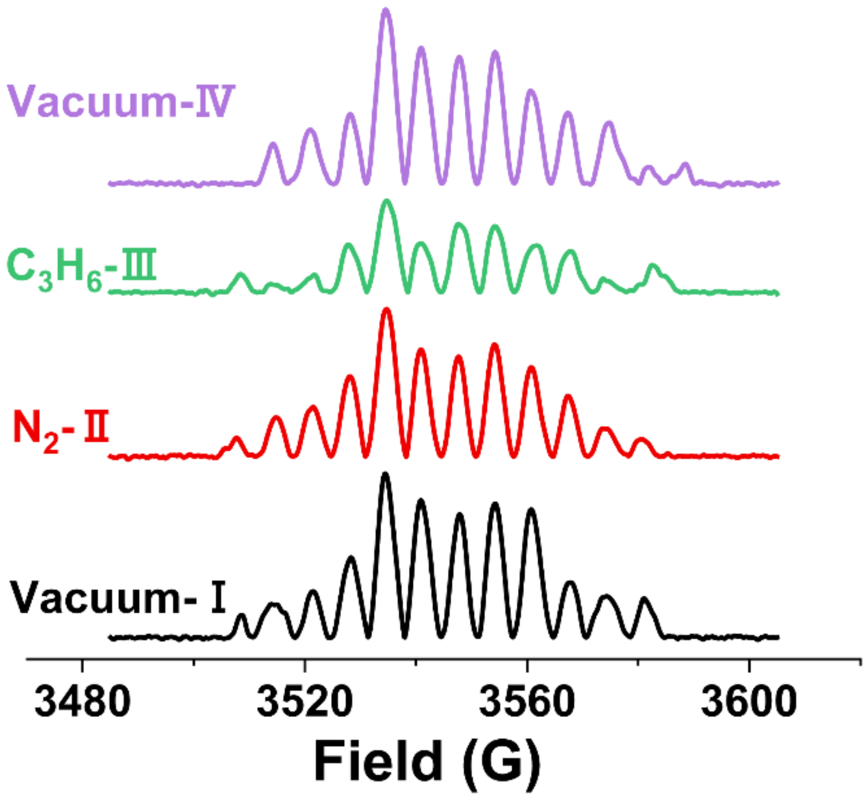

Supplementary Fig. 12 Integrated EPR spectra of $Sc_3C_2@C_{80}$⊂Py-COF under different conditions of vacuum, N_2 adsorption, and C_3H_6 adsorption.

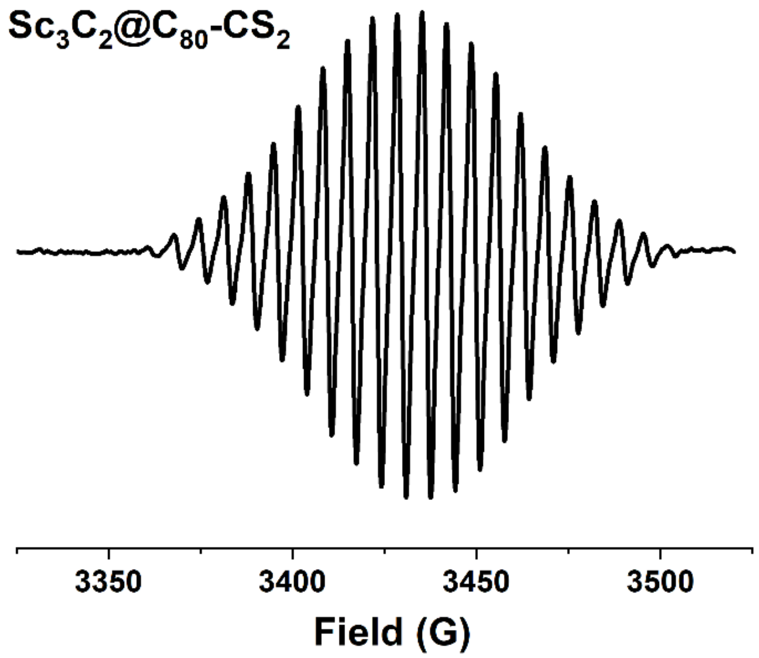

Supplementary Fig. 13 EPR spectrum of $Sc_3C_2@C_{80}$ in CS_2 solution.

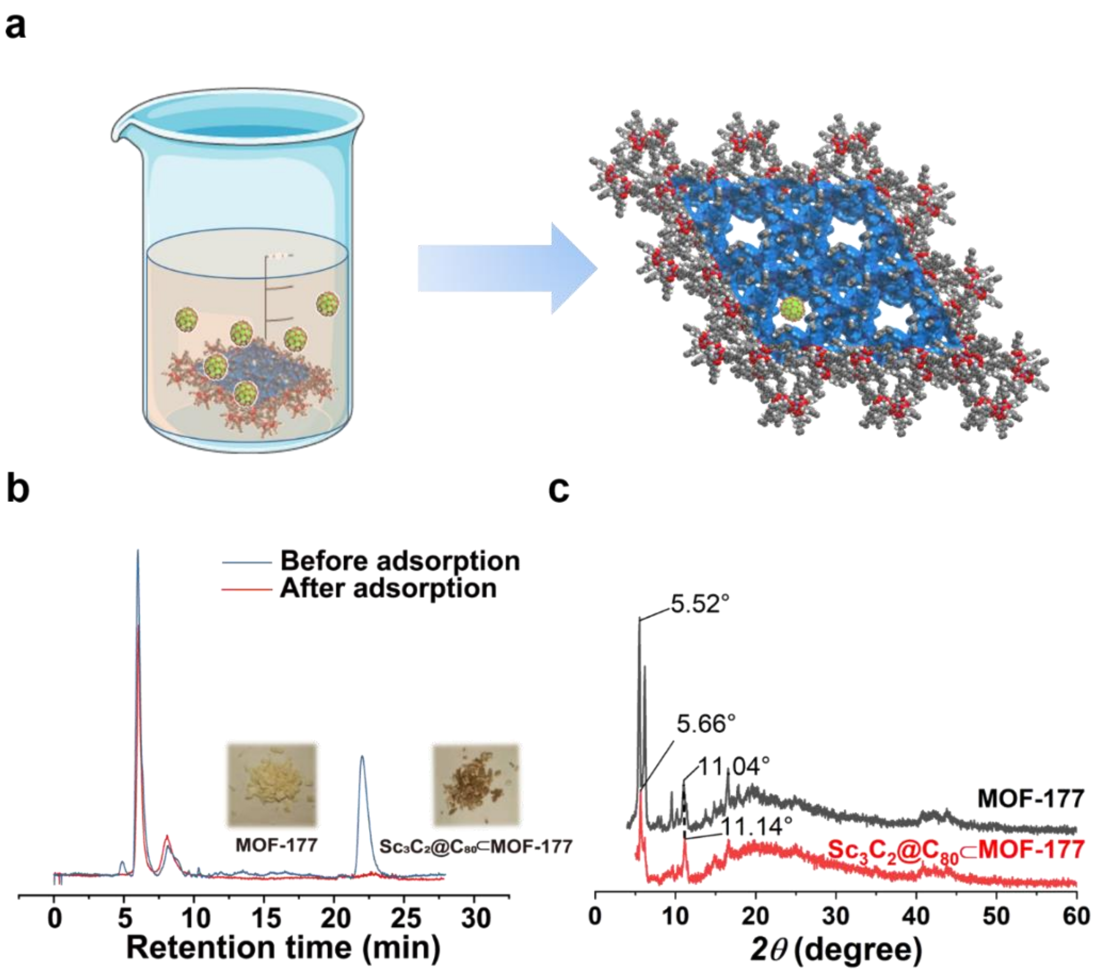

Supplementary Fig. 14 Preparation and characterizations of MOF-177 and $Sc_3C_2@C_{80}$⊂MOF-177.

a The preparation process of $Sc_3C_2@C_{80}$⊂MOF-177. **b** HPLC chromatograms of the $Sc_3C_2@C_{80}$ solutions before and after adsorption by MOF-177. The insets show the optical pictures of MOF-177 and $Sc_3C_2@C_{80}$⊂MOF-177. **c** PXRD patterns of MOF-177 and $Sc_3C_2@C_{80}$⊂MOF-177. Figure created with BioRender.com.

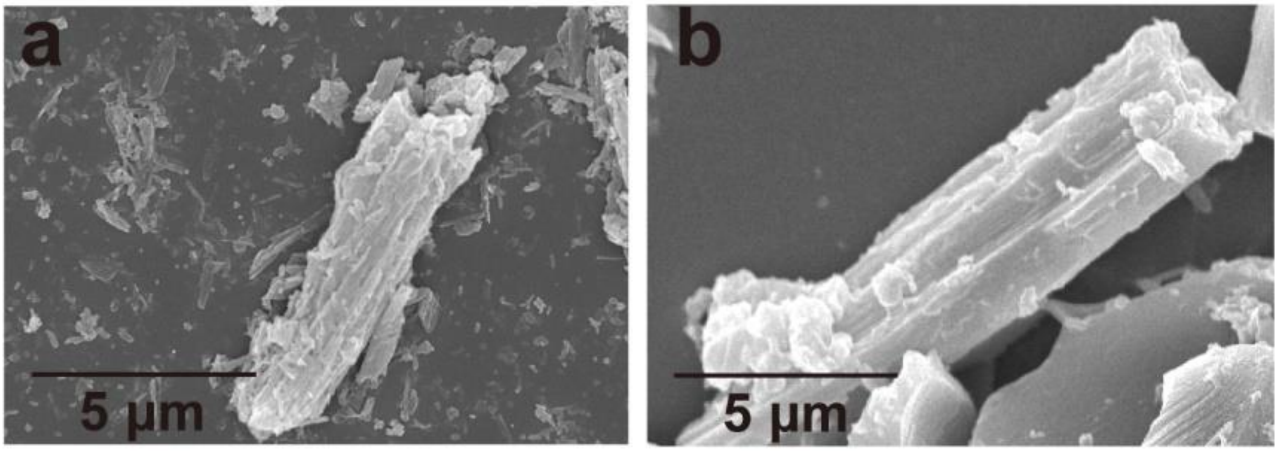

Supplementary Fig. 15 SEM images of **a** MOF-177 and **b** $Sc_3C_2@C_{80}$⊂MOF-177 complex.

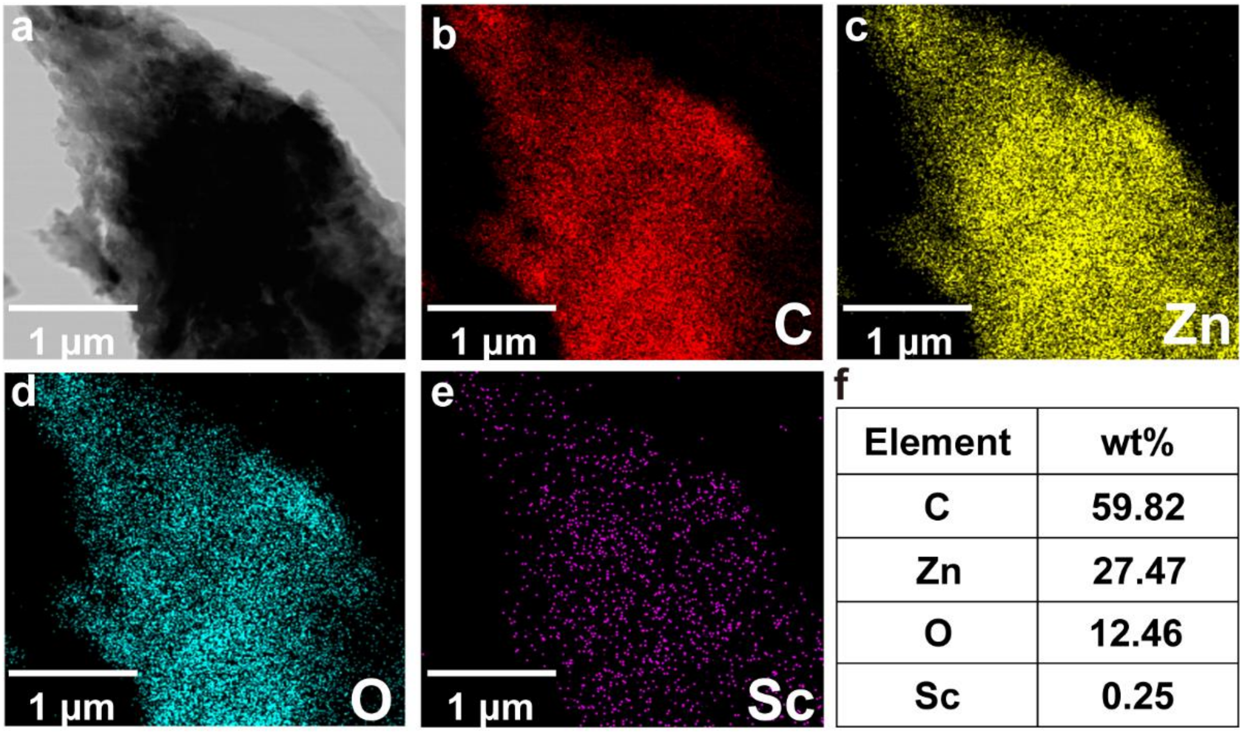

Element	wt%
C	59.82
Zn	27.47
O	12.46
Sc	0.25

Supplementary Fig. 16 TEM images of **a** $Sc_3C_2@C_{80}\subset$MOF-177 complex. TEM element mapping images of **b** C, **c** Zn, **d** O and **e** Sc for $Sc_3C_2@C_{80}\subset$MOF-177 complex. **f** Elemental fractions of $Sc_3C_2@C_{80}\subset$MOF-177 from EDS spectrum.

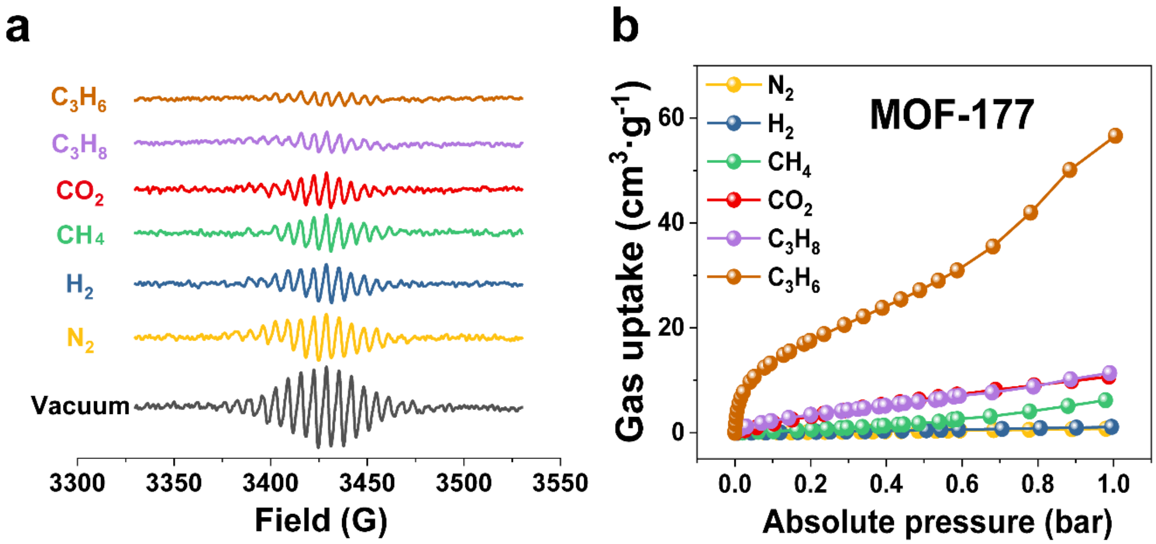

Supplementary Fig. 17 a EPR spectra of $Sc_3C_2@C_{80}$⊂MOF-177 under vacuum and after adsorbing N_2, H_2, CH_4, CO_2, C_3H_6 and C_3H_8, respectively. **b** Measured adsorption isotherms of MOF-177 for the gases of N_2, H_2, CH_4, CO_2, C_3H_6 and C_3H_8.
